# Quantification and comparison the dosimetric impact of two treatment couch model in VMAT

**DOI:** 10.1002/acm2.12206

**Published:** 2017-11-02

**Authors:** Ruohui Zhang, Yulan Gao, Wenwen Bai

**Affiliations:** ^1^ Department of Biomedical Engineering Tianjin University Tianjin China; ^2^ Department of Radiation Oncology The Fourth Hospital of Hebei Medical University Shijiazhuang China; ^3^ Department of Gastroenterology Hebei General Hospital Shijiazhuang China

**Keywords:** carbon fibre couch top, couch model, dose attenuation, Monaco

## Abstract

The use of Monte Carlo treatment planning systems (TPS) in radiation therapy has increased the dosimetric accuracy of VMAT treatment sequences. However, this accuracy is compromised by not including the treatment couch into the treatment planning process. Therefore, the impact of the treatment couch on radiation delivery output was determined, and two different couch models (uniform couch model A vs two components model B) were included and tested in the Monaco TPS to investigate which model can better quantify the couch influence on radiation dose. Relative attenuation measurements were performed following procedures outlined by TG‐176 with three phantom positions for A–B direction: on the left half (L), in the center (C) and on the right half (R) of the couch. As well as absolute dose comparison of static fields of 10 × 10 cm^2^ that were delivered through the couch tops with that calculated in the TPS with the couch model at 2 mm and 5 mm computing grid size respectively. The most severe percentage deviation was 4.60% for the phantom positioned at the left half of the couch with 5 mm grid size at gantry angle 120°. The couch model was included in the TPS with a uniform ED of 0.26 g/cm^3^ or a two component model with a fiber 0.52 g/cm^3^ and foam core 0.1 g/cm^3^. After including the treatment couch, the maximum mean dose attenuation was reduced from 3.68% without couch included to (0.60, 0.83, 0.72, and 1.02) % for model A and model B at 2 and 5 mm voxel grid size. The results obtained showed that Model A performed better than the model B, demonstrating lower deviations from measurements and better robustness against dose grid resolution changes. Considering the results of this study, we propose the systematic introduction of the couch Model A in clinical routine. All the reported findings are valid for the Elekta iBEAM ^®^ evo Extension 415 couch and these methods can also be used for other couch model.

## 1 INTRODUCTION

Patient positioning for radiotherapy is one of the most important components of the entire planning and treatment process. Carbon fiber couches are often used in external beam radiotherapy as a means of providing patient positioning. In addition to being strong, rigid, and light,^1^ carbon fiber has also been described as radiotranslucent.^2^, ^3^ Recent years the external photon beam radiotherapy delivery techniques and modalities have been undergone a rapid development, the method of radiation treatment has switched from the use of a single treatment beam to the utilization of multiple beams or rotation treatment. With the introduction of intensity modulated radiation therapy (IMRT) the number of fields used for patient treatment increases, the effect of treatment couches becomes more significant.^4^, ^5^, ^6^, ^7^, ^8^ Especially as the advanced volumetric modulated arc therapy (VMAT) delivery systems become a main role of treatment ways, which places even greater demands on delivering accuracy.

The impact of Elekta iBEAM^®^ evo Couchtop on radiation delivery has been explored by several research groups focusing on several different commercials treatment planning systems with different calculated algorithms.^9^, ^10^, ^11^, ^12^ And they reported that the pencil beam and convolution algorithms failed to accurately calculate couch attenuation at all gantry angles.

Monaco treatment planning system (TPS) employs Monte‐Carlo calculation algorithm, Shortt et al.^13^ demonstrated its high accuracy against measurements in heterogeneous geometries and which is currently routinely used as a “gold standard” against to compare analytical methods. MC simulations were performed by Teke et al. using a new DOSXYZnrc source modeled the Varian IGRT couch top. And results showed good agreement with ion chamber measurements (within 1.2%) and with TPS (within 1%).^14^ To the best of our knowledge, no Monaco (Elekta AB, Stockholm, Sweden) TPS yet implemented a iBEAM^®^ evo Extension 415 couch model for VMAT treatment. And no research on this topic has yet been carried out concerning the dosimetric impact of uniform couch model (model A, take uniform electron denstiy(ED) with 0.26 g/cm^3^) and two components model (model B, take carbon fiber shell (CFS) with ED 0.52 g/cm^3^ and foam core (FC) with ED 0.1 g/cm^3^) for VMAT treatment.

The aim of this study was to present our methods and results regarding the modeling of a carbon fiber couch (Elekta iBEAM^®^ evo Extension 415) in Monaco v.5.0. We evaluated the accuracy of the TPS in reproducing the measurements of couch attenuation by comparing measured attenuation with two model calculated attenuation. Attenuation measurements were performed following the AAPM‐TG176 guidelines.^15^ Moreover; we compared the robustness of the model A and model B against the variation in the dose grid resolution. And further the couch modeling accuracy was assessed by comparing the measured and calculated absolute average percentage deviation by the smallest and the biggest recommend calculation grid space. The paper with the aim of finding the best accurate treatment couch model accounting for any attenuation of the beam in future treatment planning to reduce the couch absorption influences.

## 2 MATERIALS AND METHODS

### 2.A Dose measurements setup

**Figure 1:**
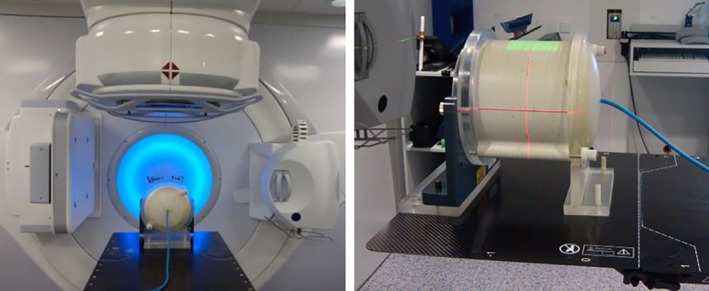
Experimental setup for direct attenuation measurements. (Phantom positioned at the couch center).

This research took place at The Fourth Hospital of Hebei Medical University, Clinical Center facilities in department of radiation oncology. Relative attenuation measurements were made following the AAPM‐TG176 guidelines.^15^ A homogeneous Cylindrical RW3 IMRT head/neck phantom model T40015 (diameter: 20 cm, length: 15 cm) (PTW Freiburg, Germany) with a 0.125cc semiflex ionization chamber (PTW30013; PTW, Freiburg, Germany) at its center was placed at the isocenter of a Elekta Synergy Linac (Elekta Oncology Systems, Crawley, UK) with MLCi2 multileaf collimator. The phantom was laterally centered on the couch, a gap of 7 cm was present between the surface of the phantom and the couch top, see Fig. 1. This mimics head and neck treatments, where the patient's head is supported by a foam headrest and does not come into direct contact with the couch. Dosimetric centering was then verified irradiating a 10 × 10 cm^2^, 6 MV (PDD10: 67.2%) field 200 MU at gantry angles of 0°, 30°, 60°, 90°, 270°, 300°, 330° (IEC scale) and accepting a maximum difference between a PTW Unidos electrometer readings within 0.1%. Using the same energy and field size, ionization readings were taken for each gantry angle from 180° to 110° with an increment of 10°. Relative attenuations were calculated using the measurement at 0° as reference.^15^, ^16^, ^17^ Attenuation was defined as eq. (1).

(1) $$\text{Attenuation}=\frac{D_{\mathrm{nc}}-D_{\mathrm{pc}}}{D_{\mathrm{nc}}}*100\%$$

where *D*_pc_ represents the dose measured with the beam passing through the treatment couch and *D*_nc_ represents the dose measured with gantry angle set for 0° while the beam did not intersect the treatment couch.

### 2.B Couch modeling in the Monaco TPS and dose calculation properties

**Figure 2:**
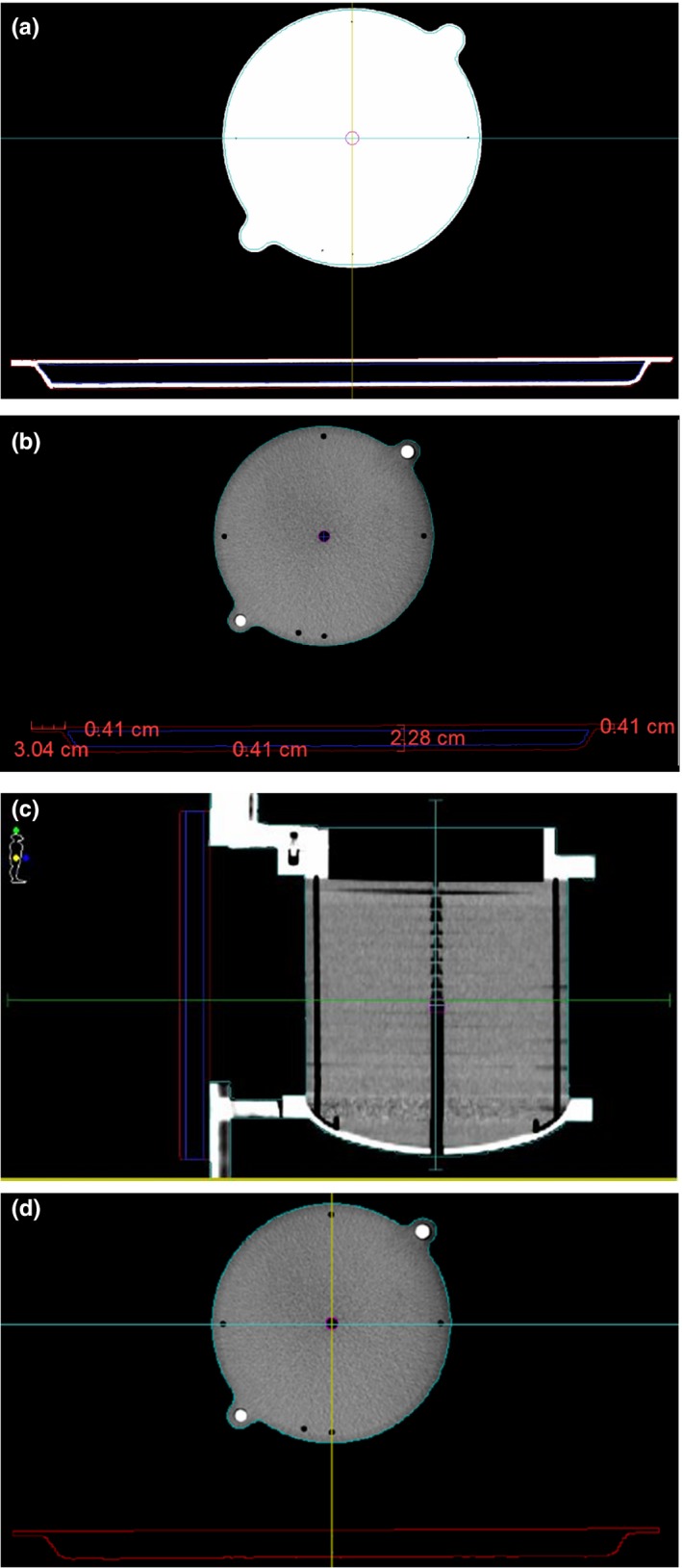
(a) Transverse CT images of the phantom positioned at couch center, (b) Contour traced iBEAM ^®^ evo Extension 415 structures from CT scan slice and modeled the couch model B, (c) Sagittal CT images of the phantom positioned at couch center, (d) modeled the couch model A.

In order to include the iBEAM^®^ evo Extension 415 in the planning system, the insert and the phantom were CT scanned with the slice thick 2 mm. The images were uploaded into the TPS (Monaco version 5.0), where the different parts of the couch top carbon fiber shell (CFS) and foam core (FC) were contoured to include them in a library for planning [Fig. 2(a)]. The active chamber volume is contoured in the TPS and calculated ‘chamber doses’ are reported as a mean dose to this volume.^14^ The Model B is constructed from a FC (assigned ED 0.1 g/cm^3^) material with a thickness of 15 mm, sandwiched between two layers of CFS (assigned ED 0.52 g/cm^3^), each with a thickness of 4 mm [Fig. 2(b)]. Model A is constructed from the contoured outline of the couch top with a thickness of 23 mm having uniform ED values of 0.26 g/cm3 [Fig. 2(d)]. For the purposes of validating the couch Model ED values in the TPS, measured and calculated couch attenuations were compared by eq. (1). Each experimental setup was first measured on the linac and then replicated at the planned in the TPS in order to mimic clinical use.

**Figure 3:**
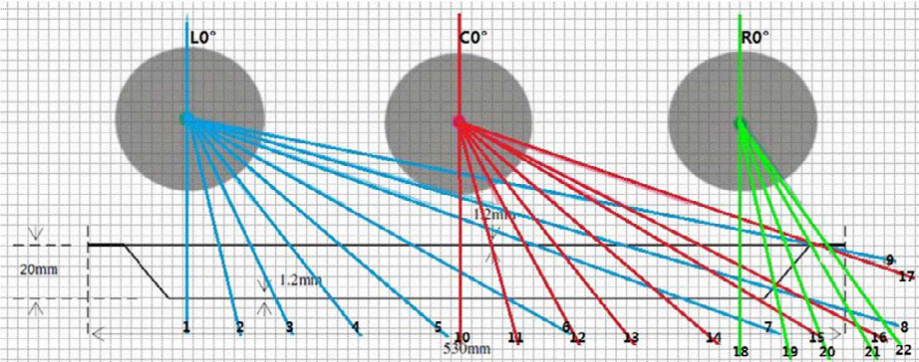
Phantom positions and Gantry angles simulated in the Monaco TPS. L: Phanton on the left half; C: Phantom was laterally centered on the couch; R: Phanton on the right half; Note: 1. L180°, 2. L170° 3. L160°, 5. L150°, 6. L140°, 7. L130° 8. L120°, 9. L116.9°; 10. C180°, 11. C170°, 12. C160°, 13. C150°, 14. C140°, 15. C130°, 16. L129.3°; 18. R180°, 19. R170°, 20. R160°, 21. R152.9°, 22. R150°.

MC simulations were performed using calculate dose to medium and the uncertainty for each simulation was kept within 0.5%. Two different calculation grid sizes of 2 mm and 5 mm were used, taking the max and min number of voxels into account. The couch modeling simulated results in the Monaco TPS were evaluated using the percentage deviation (PD) between the measured and calculated dose, defined as the follows in eq. (2), and taking the measurements dose as the reference dose.

(2) $$\text{PD}=\frac{D_{\mathrm{calculated}}-D_{\mathrm{measured}}}{D_{\mathrm{measured}}}*100\%$$

where *D*_cal._ is the calculated dose in the Monaco TPS and D_meas._ is the measured dose at the same point in the phantom. By changing assigned electron densities (ED) dialogs of couch model, to find the best electron densities for the modeled couchtop. To determine the PD for a beam perpendicular to lateral sections of the couch top,^18^ three positions of PD values were obtained as a function of gantry angle for A‐B direction: on the left half (L), in the center (C), and on the right half (R) of the couch zone (see Fig. 3). These measurements might be useful as supplements to those using oblique beams for TPS dose verification.^15^

## 3 RESULTS

### 3.A Monaco vs ionization chamber

The evaluation of beam intersection with couch components using the edge of the beam field light showed good agreement between the TPS and setup at the treatment unit. The field edge intersected a couch component within ±1° of the predicted gantry position. These measurements and simulations were all taken across half of the treatment couch; therefore any graphs only represent the attenuation spectrum from the lengthwise central axis of the couch to one side of the couch surface. However, due to the symmetry present in all the couches studied in this investigation the attenuation spectrum produced here should be representative of the spectrum for each side of the couch.

### 3.B Couchtop attenuation

**Figure 4:**
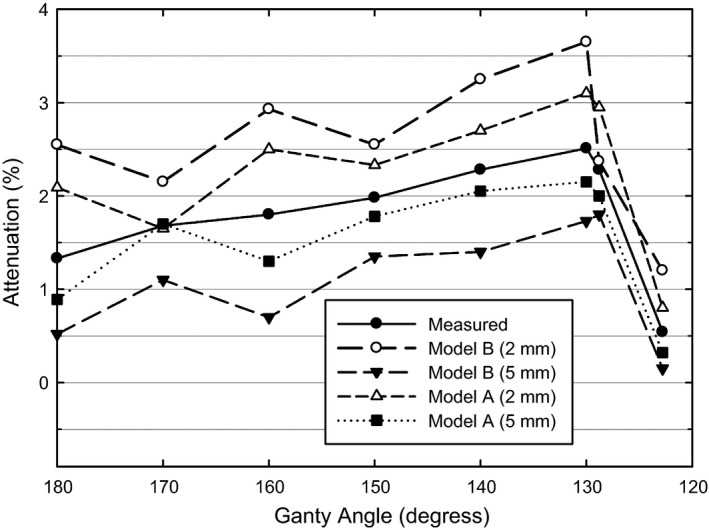
Comparison between measured and calculated attenuation profiles for the two couch model at different calculation gird space (phantom at the couch center).

Modeling the treatment couch by means of two components model B resulted in an observable overestimation or underestimation of measured attenuations for 2mm grid space and 5 mm grid space respectively. On the other hand, the uniform couch model A could be tuned in order to achieve a very good agreement with measured attenuations for each calculation grid (see Fig. 4). Couch model A profiles proved to be insensitive to dose grid variations, while couch model B showed observable differences especially for oblique beam incidence. Elekta Manual book quote the iBEAM^®^ evo Extension 415 is in perfect synergy with modern radiation therapy techniques for its low dose attenuation (only 1.5%) and providing outstanding *in situ* imaging quality and minimizing artifacts, the dose influence almost can be neglected to the patient.^19^ However, it should be noted that reported attenuation values are as per the manufacturer and the method of measurement is not specified. The attenuations we measured for the iBEAM^®^ evo Extension 415 are higher (see Fig. 4) than the Elekta declared, which they declared are only concerned with a gantry angle of 180° and thus provide little indication of the magnitude of attenuation during oblique treatments. The most couch attenuation we measured for 6MV beam energy can be reach to 2.51%, almost one point seven times of the Elekta Company declared couch attenuation, when the phantom positioned at the distal of the couch beam penetrated the longest trace of the couch the attenuation will be more serious can reach to 3.8%. If we added 2% of the TPS calculated uncertainty, and then the total uncertainty can be almost reached to 5.8%, this value is far beyond the ICRU recommended that the overall accuracy in the radiation dose delivered to the patient should be within 5%.

### 3.C Percentage deviation (PD): model A vs model B

A comparison of the percentage deviation between the measured dose and the calculated dose with and without the treatment couch modeling inserted were presented in table 1. Analysis of the PD between model A and model B shows marked improvement. The model A is better than model B, not only for the maximum PD of the single beam are 1.82% (gantry at 130°) and 1.91% (gantry at 120°), respectively, but for the mean differences between measured and calculated PD were lower for the couch Model A than the couch Model B at three phantom positions with different calculated grid spaces. These results are in accordance with the simulated couch attenuation in Monaco TPS of two couch model, see Fig. 4. Although the model A is better than model B, the ranges of observed PD were comparable for both couch Model A and Model B. Table 1 also illustrates that both model can decrease the absolute average PD from the maximum 4.60% to be within 1.82% at minimum and maximum calculated grid spacing, which within the AAPM Task Group 53 recommended acceptability criteria 2% for external beam dose calculations.^20^

**Table 1:** Measured and calculated percentage divisions of two couch model at three phantom positions with different calculation grid space

σ* *= 0.5%, Percentage Deviation (%)									
Phantom position	Couch left			Couch center			Couch right		
Gantry angle(°)	Model A	Model B	Without couch	Model A	Model B	Without couch	Model A	Model B	Without couch
Grid size: 2 mm									
180	−0.33	−0.38	3.2	−0.70	−0.82	3.18	−0.57	0.40	3.05
170	0.23	−0.80	3.08	−0.27	−0.15	3.13	−0.10	−0.75	2.99
160	−0.35	−1.10	3.10	−1.60	−1.04	3.00	−0.2	−0.80	2.99
152.9	**–**	**–**	**–**	**–**	**–**	**–**	0.15	−0.30	1.70
150	−0.27	−0.58	2.35	−0.10	−0.04	2.39	0.56	0.1	1.90
**142.2**	**–**	**–**	**–**	**–**	**–**	**–**	0.16	0.42	1.92
140	−1.02	−0.80	2.36	−0.58	−1.2	2.37	**–**	**–**	**–**
130	−0.61	0.2	3.11	−0.60	−1.44	2.80	**–**	**–**	**–**
129.3	**–**	**–**	**–**	−0.02	−0.84	3.95	**–**	**–**	**–**
**122.8**	**–**	**–**	**–**	0.92	−1.10	1.56	**–**	**–**	**–**
120	−0.97	−1.91	4.45	**–**	**–**	**–**	**–**	**–**	**–**
116.9	−0.24	−0.95	3.64	**–**	**–**	**–**	**–**	**–**	**–**
**113.2**	−0.55	−0.07	1.87	**–**	**–**	**–**	**–**	**–**	**–**
Absolute Ave. PD	0.51	0.75	3.01	0.60	0.83	2.80	0.29	0.46	2.43
Grid size: 5 mm									
180	−1.38	1.60	3.75	0.82	1.3	3.76	−0.14	−0.60	3.66
170	−0.64	−0.20	3.77	0.14	0.46	3.78	−0.22	−0.36	3.67
160	0.40	−0.20	2.85	0.95	0.92	2.75	−0.42	0.65	2.68
152.9	**–**	**–**	**–**	**–**	**–**	**–**	0.09	0.83	2.28
150	−0.27	−1.76	3.35	0.8	1.52	3.45	0.85	0.16	2.97
**142.2**	**–**	**–**	**–**	**–**	**–**	**–**	0.27	1.40	0.99
140	0.23	1.32	3.92	0.21	−0.15	4.00	**–**	**–**	**–**
130	0.20	0.71	4.3	−1.82	0.9	3.99	**–**	**–**	**–**
129.3	**–**	**–**	**–**	−0.25	0.95	0.22	**–**	**–**	**–**
**122.8**	**–**	**–**	**–**	−0.66	1.15	1.07	**–**	**–**	**–**
120	−1.06	−0.87	4.60	**–**	**–**	**–**	**–**	**–**	**–**
116.9	−0.35	0.85	4.53	**–**	**–**	**–**	**–**	**–**	**–**
**113.2**	0.60	−1.73	2.10	**–**	**–**	**–**	**–**	**–**	**–**
Absolute Ave. PD	0.57	1.02	3.68	0.72	0.92	2.87	0.33	0.67	2.87

Comments: The bolded gantry angles (142.2°, 122.8°, 113.2°) were the field isocenter exactly penetrate the couch edge, which were used to validating the couch position in the Monaco TPS, and the calculated absolute average percentage deviation value not include these value; the meaning of signal ‘**–**’, indicate no measured at that angle.

## 4 DISCUSSION

To our knowledge, the best method to take into account the beam attenuation and dosimetric aspects of the iBEAM evo couchtop is using identical couchtop both for patient CT scan and treatment. The iBEAM evo CT Overlay is identical in design, geometry and dosimetric properties with the iBEAM evo EP (main couchtop) and rests above the plane of the original CT cradle. By utilizing the same tabletop configuration for planning and treatment, both patient position and beam modeling can be accurately represented and replicated, but it is unrealistic for the extension parts of iBEAM^®^ evo Extension 415. McCormack et al.^21^ proposed a “simple” solution using a correction factor based on the couchtop attenuation to adjust the beam's MU to account for a fixed posterior oblique beam. This way can easily execute on conventional 2D and 3D‐CRT planning, but for the IMRT and VMAT treatment modality they are delivered by a series of different weighted sub segments to achieve certain dosimetric objectives, it is almost impossible for them to use this ways. However, this process may not accurately predict the treatment couch's attenuation properties. For beams intersection with the edge of the couchtop and only partially filed size are attenuated by the couchtop, adjusting the MU by the correction factor may result in a dose increase to the volume irradiated by the unattenuated portion of the field. Therefore, simply adjusting the beam's MU based on the attenuation factor at iBEAM evo Couchtop may result in an underestimated or overestimated dose distribution at the distal or proximal periphery of the beam.

In this study, we developed two couch model in Monaco TPS for the couchtop of iBEAM^®^ evo Extension 415 and evaluated its effectiveness in account for the beam intersection with the couchtop attenuation. From the figure 4 and Table 1 which can be known that for the iBEAM^®^ evo Extension 415 couch model using the uniform couch model A with ED 0.26 g/cm^3^ can obtained the best agreement between measured and Monaco TPS calculated doses than two components model B. The maximum PD of the single beam was 1.82%, this value were better than Venselaar, Welleweerd and Mijnheer^22^ suggested of the TPS generally accepted tolerance is 2% for 2 mm grid space. And this result is similar or better than the reported results achieved with different methods of couch incorporation in a commercial TPS.^9^, ^12^, ^16^, ^17^, ^23^

Our test shows that the best fit ED of two components for model B are in agreement with findings by Smith et al.^10^ who modeled the iBEAM evo carbon fiber couch in CMS XiO v4.3 (CMS Inc., Saint Louis, MO, USA) and Nucletron OMP v3.1(Nucletron BV, Veenendal, the Netherlands) TPS. These densities are lower than the actual shell density (1.2 g/cm^3^)^12^ to compensate for the increased thickness (4 mm) of the CFS compared to its real counterpart (1.2 mm). Moreover, in the Monaco TPS, before dose calculation structures must be converted to 3D voxel grid.^24^ And Monaco needs to determine what percentage of a voxel is included as part of the structure when only a portion of the voxel falls inside the structure. Due to this fact, for the two components model B, voxels across the CFS experience a relevant partial volume effect with the surrounding low density media (air or foam). This leads to the underestimation of the CFS density, which results in underestimated values for the calculated attenuations, compared to the measurements, see Fig. 4. While as voxels across the FC experience a relevant partial volume effect with the surrounding high density media (CFS). This leads to the overestimation of the FC density, which results in overestimated values for the calculated attenuations, compared to the measurements. The structure of the couch Model A is a geometrical entity with the same ED, which is not affected by the aforementioned issues. This represents an advantage of the model A over the model B.

The observed robustness against the dose grid resolution constitutes an additional advantage of couch Model A over the couch Model B. Once again this may be due to the “voxelized” influence and partial volume effect. As described above, Model B consist of a constant 4 mm thick CFS surrounding and an internal FC homogeneous structure. It can be expected that by changing the dose grid resolution from 2 × 2 × 2 mm^3^ to 5 × 5 × 5 mm^3^, different densities are sampled near the CFS due to a different number of voxels averaging with the surrounding low density materials. This fact leads to poor robustness against dose grid resolution of the couch Model B. Since Model A have a structure with uniform ED, which is much less affected by this issue.

The one disadvantage of the couch model inserted is that it increased the TPS calculation time as the calculated space volume will be increased when the couch model was included. The calculation time without and with couch model included were 5 min and 8 min for calculated grid space 2 mm, respectively, almost increased by 60%. Another point that should be taken notice of is the incorporation of the iBEAM^®^ evo couchtop into the TPS which relies on accurate patient positioning with respect to the couch center, which require indexed immobilization devices to constrain setup variation, both longitudinally and laterally. This is because the left to right shifts in patient position will result in beam path length in the couch being different and results in different degree of attenuation. Mihaylov et al.^25^ reported that the dose differences become larger than 2% for the 6 MV photons when lateral couch displacement is in excess of ±5 cm. So it is important that some form of indexing patient positioning devices be implemented.

Future research should be done to include the couch model we have modeled into more clinical patient CT sets (e.g. head and neck patients and abdomen patient) in the Monaco treatment planning system to evaluate the couchtop influence on the patient target dose delivered with VMAT and IMRT. And the skin dose delivered through an iBEAM^®^ evo Couchtop and its variability with an angle of beam incidence should be further investigated.

## 5 CONCLUSIONS

Attenuation of the Elekta iBEAM^®^ evo Extension 415 couch was modeled in the Monaco TPS which currently supports automatic incorporation of patient support devices. Two couch models were tested: (i) one with uniform ED Model (model A) and (ii) one with two components model (model B) in the planning CT. Both model found absolute average deviations within 1.02% with respect to the measurements, demonstrating the ability of TPS for modeling the treatment couch attenuation. For several situations, Model A performed better than the model B, demonstrating lower deviations from measurements and better robustness against dose grid resolution changes. Optimized materials and densities for modeling the Elekta iBEAM^®^ evo Extension 415 couch with Model A are provided. Considering the results of this study, we propose the systematic introduction of the couch Model A in clinical routine. All the reported findings are valid for the Elekta iBEAM^®^ evo Extension 415 couch. However, these methods can also be used for other couch model; the user can contour their own couches to include them in a library for planning.

## ACKNOWLEDGMENTS

The authors thank the Hebei Provincial Medical Science Foundation of China (20130253). And this study was partially supported by a grant Project Funded by the Introduction of Overseas Students of Hebei Province (C2015005006). The authors also thank Professor Feng and Chi for writing assistance. The authors also thank the editor who gave our article a chance to be published.

## CONFLICT OF INTEREST

The authors declare no conflict of interest.
